# On the Causes of Rapid Diversification in the Páramos: Isolation by Ecology and Genomic Divergence in *Espeletia*

**DOI:** 10.3389/fpls.2018.01700

**Published:** 2018-12-03

**Authors:** Andrés J. Cortés, Luz N. Garzón, Jhon B. Valencia, Santiago Madriñán

**Affiliations:** ^1^Department of Biological and Environmental Sciences, University of Gothenburg, Gothenburg, Sweden; ^2^Escuela de Biología, Universidad Industrial de Santander, Bucaramanga, Colombia; ^3^Facultad de Ingeniera y Administracin, Universidad Nacional de Colombia - Sede Palmira, Palmira, Colombia; ^4^Departamento de Ciencias Biológicas, Universidad de los Andes, Bogotá, Colombia; ^5^Jardín Botánico de Cartagena “Guillermo Piñeres”, Turbaco, Colombia

**Keywords:** isolation-by-distance (IBD), isolation-by-environment (IBE), genome–environment associations (GEA), genomic signatures of selection, GBS-derived SNP markers, neotropical alpine region

## Abstract

How diversity arises and what is the relative role of allopatric and ecological divergence are among the most persistent questions in evolution and ecology. Here, we assessed whether ecological divergence has enhanced the diversification of the Neotropical alpine plant complex *Espeletia*, also known as frailejones. This genus has one of the highest diversification rates ever reported and is distributed in the world’s fastest evolving biodiversity hotspot, the Páramo (Neotropical alpine grasslands at elevations of c. 2800–4700 m). Our goal was to determine whether ecology plays a role in divergence within the *Espeletia* complex by quantifying genome-wide patterns of ecological divergence. We characterized 162 samples of the three most common and contrasting ecotypes (distinct morphotypes occupying particular habitats) co-occurring in six localities in the northern Andes using Genotyping by Sequencing. Contrasting ecotypes were caulescent cloud forest populations, caulescent populations from wind-sheltered and well-irrigated depressions and acaulescent populations from wind-exposed drier slopes. We found high polymorphism with a total of 1,273 single nucleotide polymorphisms (SNPs) that defined the relationships among nine genetic clusters. We quantified allelic associations of these markers with localities and habitats using 18 different general and mixed-effects statistical models that accounted for phylogenetic distance. Despite that these models always yielded more SNPs associated with the localities, markers associated with the habitat types were recovered too. We found strong evidence for isolation-by-distance (IBD) across populations despite rampant gene flow, as expected for plant groups with limited seed dispersal. Contrasts between populations of different habitat types showed that an isolation-by-environment (IBE) trend emerged and masked the IBD signal. Maximum likelihood estimation of the number of migrants per generation (N_e_m) among ecotypes confirmed the IBE pattern. This result illustrates the importance of mountains’ environmental variation at a local scale in generating rapid morphological radiations and maintaining multiple adaptations in a fast-evolving ecosystem like the Páramo.

## Introduction

Diversification is recognized as an important process generating phenotypic and genetic variation in plants and animals. However, its relationship with ecological variation and genomic and morphological divergence is just starting to be understood (Nosil and Feder, 2011; Strasburg et al., 2011). For instance, despite ecological-driven diversification often being considered rare (Coyne and Orr, 2004), it has progressively been acknowledged as a main driver of diversification (Schluter, 2001, 2009; Rieseberg and Willis, 2007; Nosil and Harmon, 2009; Nosil, 2012). Yet, the consequences of ecological divergence at the genetic level remain poorly documented (Gompert et al., 2014). Rapidly evolving clades from highly heterogeneous ecosystems may bridge this gap since they offer a good set up to explore the genomic patterns associated with ecological variation during divergence with gene flow, ultimately leading to insights into how organisms adapt and diversify (Stinchcombe and Hoekstra, 2008; Hoffmann and Sgro, 2011; Savolainen et al., 2013).

The Páramo, the world’s fastest evolving biodiversity hotspot (Madriñán et al., 2013), is an alpine ecosystem dominated by species-rich grasslands above the treeline in the American tropics (at elevations of ca. 2800–4700), that despite its small surface area (35,000 km^2^), may contain over 3,000 plant species, many of which are found nowhere else on the planet (Luteyn, 1999; Hughes and Atchison, 2015). Plant groups that occupy these ‘sky islands’ (Sklenář et al., 2014) are therefore good candidates to explore ecological divergence because they are a likely product of unique adaptations to an extreme environment that evolved during the last five million years when the Andes reached an altitude that was capable of sustaining this type of vegetation (Antonelli et al., 2009; Hoorn et al., 2010; Madriñán et al., 2013). Three hypotheses have been proposed to explain the unparalleled diversification rate in the Páramo. First, Pleistocene glacial cycling coupled with the Andean uplift during the last 2.4 Myr led to repeated periods of connectivity and spatial isolation, which is thought to have generated many taxa in a short period – the ‘species pump hypothesis.’ Second, even though allopatric diversification may have been the main cause of isolation, the high levels of ultraviolet (UV) light of the high tropical mountains may have induced a rapid mutation rate (Davies et al., 2004; Willis et al., 2009) and in turn promoted morphological differentiation and even reproductive isolation. Third, it is also possible that environmental heterogeneity and ecological opportunity were the major factors driving rapid diversification. Mountains’ local scale environmental variation helps maintaining adaptation, which in turn can trigger the isolation needed for ecotypes to evolve into new species (Bridle and Vines, 2007; Cortés, 2013, 2015; Cortés et al., 2014; Cortés and Blair, 2018b; Cortés and Wheeler, 2018).

Here, we examine the iconic *Espeletia* complex, an appropriate model because it is one of the most rapidly evolving plant groups (Madriñán et al., 2013). *Espeletia* species (ca. 120) are ecologically abundant in the Páramo (Luteyn, 1999). *Espeletia* likely originated in the Venezuelan Andes (Pouchon et al., 2018) from where it spread southward through the Colombian Eastern Cordillera to the Ecuadorian Andes, followed by a northward colonization of the Colombian Central and Western Cordilleras (Cuatrecasas, 2013). The phylogeny of the genus is largely unresolved (Diazgranados and Barber, 2017) and represents a network due to weak species boundaries (Pouchon et al., 2018) and massive hybridization (Rauscher, 2002), likely consequences of its rapid diversification. The predominant pattern of genetic differentiation among *Espeletia* populations is isolation-by-distance (IBD; Diazgranados and Barber, 2017; Padilla-GonzáLez et al., 2017), indicating allopatric divergence reinforced by limited seed dispersal. *Espeletia* plants are ecologically heterogeneous, with populations adapted to grow in the wet depressions of high valleys, in the dry exposed slopes, or even within the forests at the tree line, therefore experiencing a wide range of climatic conditions within elevations and localities. Despite the heterogeneity in the habitat types, isolation-by-environment (IBE), in which genetic and environmental distances are positively correlated independent of geographic distance, has never been tested explicitly within this system, as has been partially envisioned for other Páramo genera such as *Lupinus* (Hughes and Eastwood, 2006; Contreras-Ortiz et al., 2018), *Loricaria* (Kolar et al., 2016) and *Senecio* (Duskova et al., 2017).

Genomic studies in plant populations have proven that genome–environment associations (GEA) are useful to identify genomic signatures of IBE, which are the genetic association with environmental variables. Mostly, these studies have associated single-nucleotide polymorphism (SNP) alleles and parameters from the environment of origin to infer genetic adaptive variants. For instance, Turner et al. (2010) and Fischer et al. (2013) predicted genetic adaptive variation to serpentine soils and to topo-climatic factors in *Arabidopsis lyrata* and *A. halleri*, respectively, Hancock et al. (2011) captured climate-adaptive loci in a set of geographically diverse *A. thaliana*, Yeaman et al. (2016) found convergent adaptation in two species of conifers, Pluess et al. (2016) detected local adaptation to climate at a regional scale in *Fagus sylvatica*, and Cortés and Blair (2018a) found evidence for disruptive selection on drought tolerance in wild *Phaseolus vulgaris*. Since the GEA approach is a robust strategy for characterizing the genomic landscape of IBE and discovering genetic sources of adaptive variation, in this study we couple GEA with an in-depth sampling of *Espeletia* ecotypes across the three contrasting habitats typically observed in the Páramo, which are: the upper limit of the wet cloud forest, the well-irrigated depressions of the high valleys, and the more wind-exposed and drier slopes. Abiotic stresses such as frost and flooding are known to vary among these habitats, as well as the plant traits (e.g., plant height, pubescence, presence of aerenchyma) that likely confer adaptation to this assortment of environmental conditions (Monasterio and Sarmiento, 1991; Sklenář et al., 2010a,b, 2012, 2016).

By combining a sampling spanning the three habitats where *Espeletia* is typically found across several representative localities of the genus’ distribution in the Central and Eastern Cordilleras of the northern Andes, with a whole-genome genotyping method such as Genotyping by Sequencing (GBS, Elshire et al., 2011), we aimed in this study to (1) quantify the genome-wide patterns of geographic and ecological divergence among *Espeletia* populations and ecotypes, and (2) compute SNP allelic associations with localities and habitats in order to identify sources for genetic isolation and adaptive variation. We hypothesized that if ecological differentiation contributed to genetic divergence in the *Espeletia* complex, then we should be able to recover for the first time more subtle and localized signals of IBE, besides a predominant pattern of IBD.

## Materials and Methods

### Sample Collection, DNA Extraction and Genotyping-by-Sequencing

**FIGURE 1:**
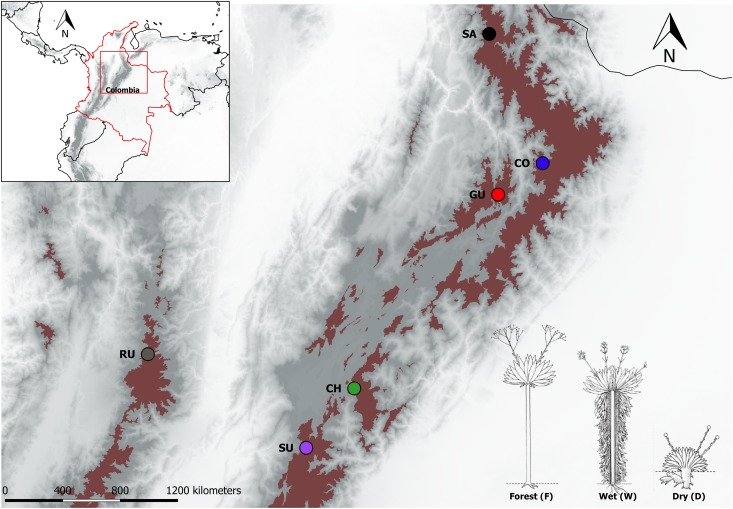
Páramo localities in the northern Andes sampled in this study. Red shaded areas mark the 2013 Páramo delimitation in the Colombian Andes as taken from http://paramo.uniandes.edu.co/. Localities are identified by different colors and abbreviated as follows: Chingaza (CH), Cocuy (CO), Guantiva (GU), Santurbán (SA), Sumapaz (SU), and Ruíz (RU). In the bottom right, a schematic drawing of the different ecotypes according to Cuatrecasas (2013), are coded as follows: caulescent populations from the cloud forest (F), caulescent populations from wind-sheltered well-irrigated depressions (W) and acaulescent populations from wind-exposed drier slopes (D).

A total of 162 individual plants from the *Espeletia* complex were used in this study (Figure 1 and Supplementary Table S1). These individuals comprised 17 different taxonomic groups (*Espeletia grandiflora, E. uribei, E. killipii, Espeletiopsis colombiana, E. lopezii, Espeletiopsis jimenez-quesadae, E. boyasensis, E. congestiflora, E. arbelaezii, E. discoidea, E. hartwegiana, E. estanislana, E. standleyana, E. conglomerata, E. argentea, E. cabrerensis, E. summapacis*, from a total of ca. 120, according to Cuatrecasas (2013), although this taxonomy is likely to change, as proposed by Pouchon et al. (2018), because of weak species boundaries and reiterative paraphyly) and were chosen to be representative ecotypes of the three most common habitats: caulescent populations from the cloudy forest (F) and from wind-sheltered well-irrigated depressions (W), and acaulescent populations from wind-exposed drier slopes (D), co-occurring in six localities in the northern Andes (Ruíz in the Colombian Central Cordillera, and Santurbán, Cocuy, Guantiva, Chingaza and Sumapaz, from north to south, in the Colombian Eastern Cordillera). Two taxonomic groups were found in the wind-exposed drier slopes of Guantiva, so there was a total of 19, rather than 18 (6 × 3), locality–habitat combinations, hereinafter referred to as populations. This sampling is unique because the chosen populations merge properties associated with geographic as well as ecological isolation. Sampled leaves were shaved using a field knife in order to improve drying in silica gel. Genomic DNA was extracted using the QIAGEN DNeasy Plant Mini Kit (QIAGEN, Germany). DNA concentration was quantified in a Qubit^®^ dsDNA HS Fluorometer (Life Technologies, Sweden). Two 96-plex genotyping-by-sequencing (GBS) libraries were performed with *Ms*II digestions according to Elshire et al. (2011) and sequenced at LGC Genomics (Berlin, Germany).

### Read Pre-processing, GBS Clustering, Alignment and SNP Discovery

Demultiplexing, cleaning, and filtering of Illumina reads was performed with the Illumina bcl2fastq v. 2.17.1.14 software (Illumina, San Diego, CA, United States). A total of 1 or 2 mismatches or Ns were allowed in the barcode read when the barcode distances between all libraries allowed for it. Demultiplexing of library groups into samples was done according to inline barcodes and verification of restriction site. No mismatches or Ns were allowed in the inline barcodes but Ns were allowed in the restriction site. Restriction enzyme site filtering of read 5′ ends was carried out so that reads with 5′ ends not matching the restriction enzyme site were discarded. Quality trimming of adapter-clipped reads was done by removing reads containing Ns, trimming reads at 3′-end to get a minimum average Phred quality score of 20 over a window of ten bases and discarding reads with final length < 20.

Alignment and clustering of combined reads was performed with CD-HIT-EST v. 1^1^, allowing up to 5% difference. Alignment of subsampled quality-trimmed reads against all clusters was done using BWA (Li and Durbin, 2007) v. 0.7.12^2^, resulting in a single combined alignment for all samples in a coordinate-sorted BAM format. Variant discovery and genotyping of samples was done with Freebayes v. 1.0.2-16^3^, allowing for a minimum base quality and a minimum supporting allele qsum of 10, and a read mismatch limit of three. Filtering of variants was done using a GBS-specific rule set with >5 read count for a locus, >5% minimum allele frequency (MAF) across all samples, and an observation of genotype frequency of at least 72% (116 samples). The filtered dataset was inspected with TASSEL v. 3.0 (Glaubitz et al., 2014), resulting in a final set of 1,273 SNPs.

### Overall Patterns of Genetic Isolation

We examined broad patterns of population structure using principal coordinates analysis (PCoA) implemented in Trait Analysis by aSSociation, Evolution and Linkage v.5 (Bradbury et al., 2007). The construction of customized PCoA diagrams was carried out in R v.3.3.1 (R Core Team). Population structure was further examined by an AMOVA test according to Excoffier et al. (2005), and by allowing admixture through a Bayesian analysis using STRUCTURE (Pritchard et al., 2000) on LD-free sites (*R*^2^ = 0.0648 ± 0.0002, CI 95%: 0.003 – 0.625). Five independent runs for each *K* value from *K* = 2 to *K* = 19 (the total number of expected populations) used an admixture model with 100,000 burn-ins and 200,000 iterations in the MCMC analysis. A bar graph of the results was generated for each *K* value using CLUMPP (Jakobsson and Rosenberg, 2007). The optimal *K* value was determined based on the PCoA diagrams, cross-run cluster stability and likelihood of the graph model following Evanno et al. (2005). Finally, in order to compute phylogenetic distances a phylogenetic tree was inferred using the program SNAPP 1.3.0 (Bryant et al., 2012) included in the package BEAST 2.4.5 (Bouckaert et al., 2014). We used all 19 populations as *a priori* designated clusters. The analysis was run for 1,000,000 generations sampling every 1,000 generations. The log files were evaluated for convergence with the program Tracer 1.5 (Drummond and Rambaut, 2007) and trees in the 95% highest posterior density (HPD) set were analyzed with SNAPP-TreeSetAnalyser 2.4.5 using 10% of topologies as burn-in. Resulted tree files (cloudgrams) were visualized using DensiTree (Bouckaert, 2010). A consensus tree was rooted using the populations from Santurbán, following Diazgranados and Barber (2017).

In order to explore subtle divergence patterns among populations and ecotypes, the entire dataset was partitioned by habitats and localities, so that the habitat-based dataset included populations from the Cocuy and Guantiva localities, whereas the locality-based dataset included populations from the remaining localities. SNP markers were filtered for each of these new datasets using the same MAF and missing data rules than for the full dataset (see previous section), leading to 897 and 812 SNP markers, respectively. This partition led to three different datasets (entire, locality-based and habitat-based datasets) that were used in subsequent analyses. Four reiteratively misplaced samples led to an acceptable error rate (Glaubitz et al., 2014) of 2.5% and were excluded from further analyses.

### Allelic Associations With Localities and Habitats

In order to perform GEA analyses between the SNP markers and the localities, and between the SNP markers and the habitats, we used the software Trait Analysis by aSSociation Evolution and Linkage. For these GEA analyses the habitats were ranked according to the expected, hereinafter referred to as theoretical, exposure to frost and soil moisture content, as coded in Supplementary Table S1. Six generalized (GLM) and mixed (MLM) linear models were compared for each of the three datasets for a total of 18 models. Within each model family, three models were built as follows: (1) model with the locality as a fixed effect, (2) model with the theoretical exposure to frost as a fixed effect and (3) model with the theoretical soil moisture content as a fixed effect. All models included the phylogenetic distance computed from the phylogeny as a covariate. All MLMs used an IBS kinship matrix as a random effect to control for genomic background implementing the EMMA and P3D algorithms to reduce computing time (Zhang et al., 2010). Localities and habitats were never considered as random effects, as is tradition in the majority of association scans, because our main purpose was to detect allelic associations precisely with these factors. QQ-plots of the *P*-values were inspected to assess whether excessive numbers of false positives were generated and choose in this way the optimum model. Significant associations were determined using a strict Bonferroni correction of *P*-values at α = 0.05, leading to a significance threshold of 3.9 × 10^-5^, 6.2 × 10^-5^, and 5.6 × 10^-5^ (0.05 divided by the number of markers, 1,273, 812, and 897) or -log_10_ of 4.4, 4.2, and 4.6, for the full, locality-based and habitat-based datasets, respectively.

### Patterns of Gene Flow and Divergence Among Localities (IBD) and Habitats (IBE)

A single genome can exhibit heterogeneous patterns of geographic (IBD) and ecological (IBE) differentiation (Strasburg et al., 2011). Therefore, in order to understand the landscape of divergence in the *Espeletia* complex, the entire dataset and the contrasting datasets for localities and habitats were further subdivided according to the allelic associations found in the previous section, so that SNP markers were retained if they were significantly associated with the locality, the theoretical exposure to frost or the theoretical soil moisture content. Since the full marker set was also maintained in each case, this partition series led to a total of eleven datasets. The dataset that only included contrasting populations for localities and associated markers with the theoretical soil moisture content had just one polymorphic site and was excluded from the rest of the analyses. Pairwise *F*_ST_ values, according to Weir and Cockerham (1984), were computed in each of the eleven datasets using customized R scripts. Bidirectional gene flow among pairs of populations was then estimated as the number of migrants per generation (N_e_m) following Beerli and Felsenstein (1999), which is a highly robust maximum likelihood estimation coupled with a coalescent framework. Networks depicting pairwise bidirectional migration rates across all datasets were drawn using the R package “qgraph” (Epskamp et al., 2012). Even though more sophisticated methods have been developed to estimate gene flow, the conceptual clarity and robustness of *F*_ST_-based methods and Beerli and Felsenstein (1999)’s approach is still undoubted.

Explicit correlations between genetic differentiation and geographic and ecological distances were obtained among populations across all eleven datasets. The genetic differentiation was computed as *F*_ST_/(1 – *F*_ST_) according to Rousset (1997). On the other hand, the geographic distance was taken from the sampling coordinates using the R package “geosphere”, whereas the ecological distance was calculated independently for the theoretical exposure to frost and the theoretical soil moisture content, as ranked in Supplementary Table S1, using an Euclidean distance measure implemented in the R function *dist*. Therefore, for each dataset three different correlations with the genetic differentiation were considered (against the geographic distance and the two ecological distances), leading to a total of 33 Mantel tests. These tests used 1,000 permutations implemented in R (R Core Team). Significant associations were determined based on a strict Bonferroni correction of *P*-values at α = 0.05 (as detailed in Table 1).

**Table 1:** Correlations between genetic differentiation, geographic distance, and ecological distance (exposure to frost and soil moisture content) among populations of *Espeletia* growing in 3 different habitats and 6 localities.

Population dataset	Marker dataset	N_PW_	SNPs	Geographic distance (Km)	Ecological distance (‘Frost’)	Ecological distance (‘Humidity’)	Bonferroni threshold
All	All	171	1273	**0.61**	0.07	0.09	0.0003
All	Associated with locality	171	172	**0.95**	0.03	0.05	0.0008
All	Associated with frost	171	39	**0.48**	0.08	0.06	0.0003
All	Associated with humidity	171	28	**0.33**	0.05	0.10	0.0003
Locality-based	All	66	812	**0.95**	0.03	0.05	0.0008
Locality-based	Associated with locality	66	156	**0.95**	0.04	0.03	0.0008
Locality-based	Associated with frost	66	2	0.37	0.01	0.12	0.0008
Habitat-based	All	21	897	0.54	**0.47**	0.36	0.0024
Habitat-based	Associated with locality	21	184	0.44	**0.48**	0.30	0.0024
Habitat-based	Associated with frost	21	123	0.46	**0.51**	0.30	0.0024
Habitat-based	Associated with humidity	21	87	0.38	**0.43**	0.32	0.0024


*The entire dataset is progressively partitioned by contrasting populations for localities and habitats, and by sets of markers significantly associated with the localities, the theoretical exposure to frost and the theoretical soil moisture content (humidity). The dataset that only includes contrasting populations for localities and markers significantly associated with the theoretical soil moisture content is not shown because lack of polymorphism (one variable site). Allelic associations of the GBS-derived SNP markers with localities and habitats were quantified using 18 different general and mixed-effects statistical models that accounted for phylogenetic distance. Only results for the optimum models are shown. N_PW_ indicate the number of pairwise comparisons. Significant correlations based on Mantel tests are set in bold. Graphical depiction is shown in Supplementary Figure S6.*

## Results

### Genetic Variation Clustered in Nine Major Groups

**FIGURE 2:**
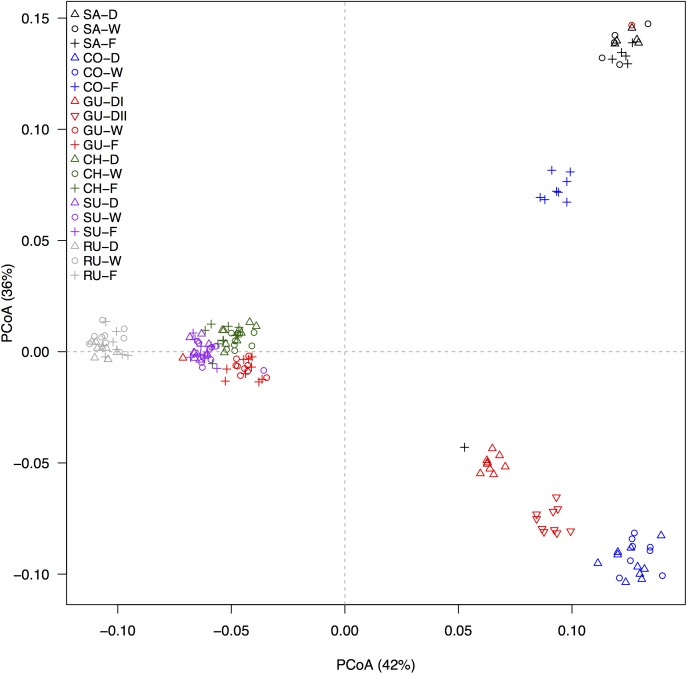
Differentiation, as revealed by principal coordinates analysis (PCoA), based on 1,273 GBS-derived SNP markers. Ecotypes are labeled by different symbols as follows: caulescent populations from cloud forest (+), caulescent populations from wind-sheltered well-irrigated depressions (o) and acaulescent populations from wind-exposed drier slopes (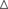), and abbreviated in the figure legend according to the habitat as forest (F), wet (W) and dry (D), respectively. Localities are identified by different colors and coded as in Figure 1. The percentage of explained variation by each axis is shown within parenthesis in the label of the corresponding axis.

The principal components analysis of 1,273 GBS-derived SNP markers recovered nine genetic clusters (Figure 2) comprising the 17 taxonomic groups and 19 populations sampled. Individuals from the localities of Ruíz and Santurbán were both independent clusters, whereas individuals from the Chingaza and Sumapaz localities clustered with the caulescent individuals from the cloud forest and the wind-sheltered well-irrigated depressions of Guantiva. The acaulescent individuals from Guantiva formed two clusters and were situated between two clusters conformed by individuals from the Cocuy locality; one of them, close to the Santurbán cluster, clustered caulescent individuals from the cloud forest, while the other grouped the rest of the individuals from Cocuy. The cluster containing individuals from the localities of Chingaza and Sumapaz and caulescent individuals from the locality of Guantiva differentiated by locality but not by habitat (Supplementary Figure S1), suggesting that all 162 individuals were clustered in a maximum of nine different genetic groupings.

**FIGURE 3:**
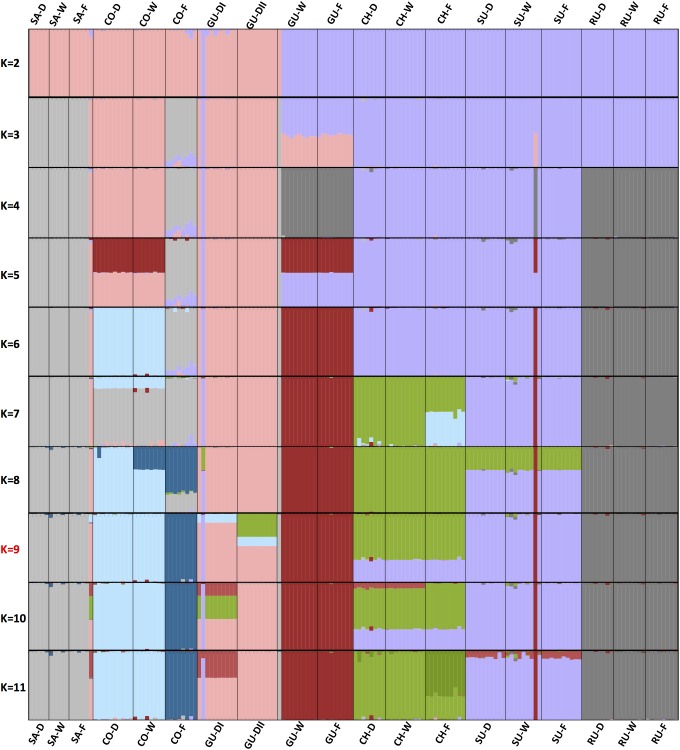
Population structure from assignment tests in STRUCTURE based on 1,273 variable SNP markers. *K*-values of 2–11 populations are shown. The optimum *K*-value (*K* = 9, Supplementary Figure S2) according to Evanno et al. (2005), the PCoA (Figure 2) and visual inspection is marked in red. Populations are sorted from north to south with the only locality from the Colombian Central Cordillera at the extreme right. The name of each population is given at the top of the bar plot and the localities and ecotypes are colored and coded as in Figure 1.

Evaluation of the population structure through unsupervised Bayesian genetic clustering allowing for admixture and implemented in STRUCTURE resulted in similar separations at *K* = 9 (Figure 3 and Supplementary Table S2), reinforcing the confirmatory observation (Rauscher, 2002; Diazgranados and Barber, 2017; Pouchon et al., 2018) of weak boundaries among taxonomic groups in the *Espeletia* complex (an AMOVA test indicated that 43.7% of the genetic variation could be explained by locality, 21.8% could be explained by habitat type within locality and 34.5% was found within ecotype). The most likely *K*-value of nine was selected based on the previous results and the increases in likelihood ratios between runs using Evanno et al.’s (2005) delta *K* statistic (Supplementary Figure S2). Separation of the populations at each *K*-value was informative. At the first level of population separation, *K* = 2, all individuals divided north–south with the acaulescent populations from Guantiva inside the northern pool. At *K* = 3 the individuals from Santurbán and the caulescent individuals from the cloud forest of Cocuy separated from the northern group. At *K* = 4 the individuals from Ruíz and the caulescent individuals from Guantiva detached from the southern group, and at *K* = 6 the latter split into an independent cluster. At *K* = 7 the population separation agreed with geographical distribution within the assembly of individuals from Chingaza and Sumapaz and the caulescent individuals from Guantiva. At *K* = 9 different levels of admixture differentiated the two acaulescent populations sampled in the wind-exposed drier slopes of Guantiva.

**FIGURE 4:**
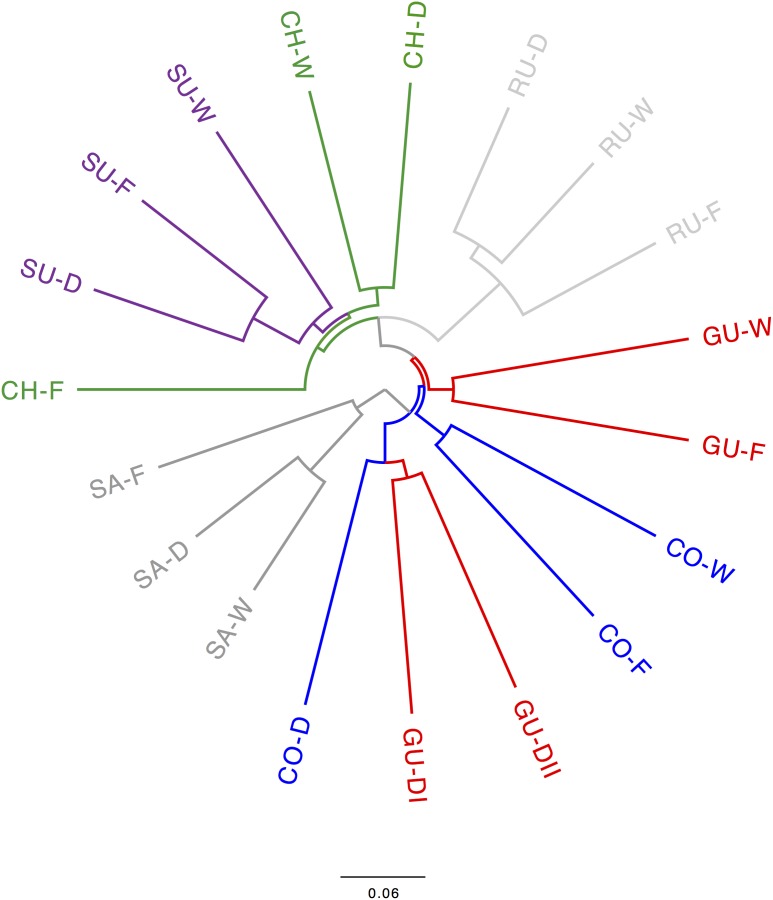
Consensus SNAPP-based phylogenetic tree of the *Espeletia* populations analyzed in the current study. Populations from Santurbán (SA) were used as outgroups for rooting the phylogenetic tree following Diazgranados and Barber (2017). Localities and ecotypes are colored and coded as in Figure 1.

The consensus SNAPP-based phylogenetic tree (Figure 4) clarified the assignment of splitting events to populations defined in STRUCTURE. Populations from the localities of Sumapaz and Ruíz were monophyletic as well as the sister clade of the latter, which comprised all populations from Chingaza and Sumapaz. A sister monophyletic clade of all these populations comprised the caulescent populations from Guantiva so that all these linages were nested within the caulescent populations from Cocuy. Acaulescent populations from Cocuy and Guantiva were assembled in a monophyletic clade, which was a sister group of the other populations. The cloudgram depicting all SNAPP-based phylogenetic trees was fully consisted with the consensus tree (Supplementary Figure S3).

### Allelic Associations With Localities Rather Than With Habitats

QQ-plots from the association analyses between the SNP markers and the localities, and between the SNP markers and the habitats (ranked according to the theoretical exposure to frost and the theoretical soil moisture content) indicated that MLM models over-controlled for population structure by using a kinship matrix, whereas GLM analyses exhibited tolerable rates of false positives (Supplementary Figure S4). These last models yielded, at a Bonferroni-corrected significance threshold of 4.4 –log_10_ (*P*-value), a total of 172, 39 and 28 SNP markers associated with the localities and with the habitats when these were ranked according to the theoretical exposure to frost and the theoretical soil moisture content, respectively (as detailed in Table 1). When only contrasting populations for localities were considered, the GLM analyses respectively yielded a total of 156, 2 and 1 SNPs associated with the localities and with the habitats when these were ranked according to the theoretical exposure to frost and the theoretical soil moisture content, according to a Bonferroni-corrected significance threshold of 4.2 -log_10_ (*P*-value). The fact that few SNP markers were associated with the habitats in this contrasting dataset for localities was consistent with a low rate of false positives in a species complex well known for its IBD pattern. On the other hand, when only contrasting populations for the habitats were considered, the GLM analyses yielded, based on a Bonferroni-corrected significance threshold of 4.6 -log_10_ (*P*-value), a total of 184, 123 and 87 SNPs respectively associated with the localities and with the habitats when these were ranked according to the theoretical exposure to frost and the theoretical soil moisture content.

### Genomic Signatures of Divergence Recover IBE Besides IBD

**FIGURE 5:**
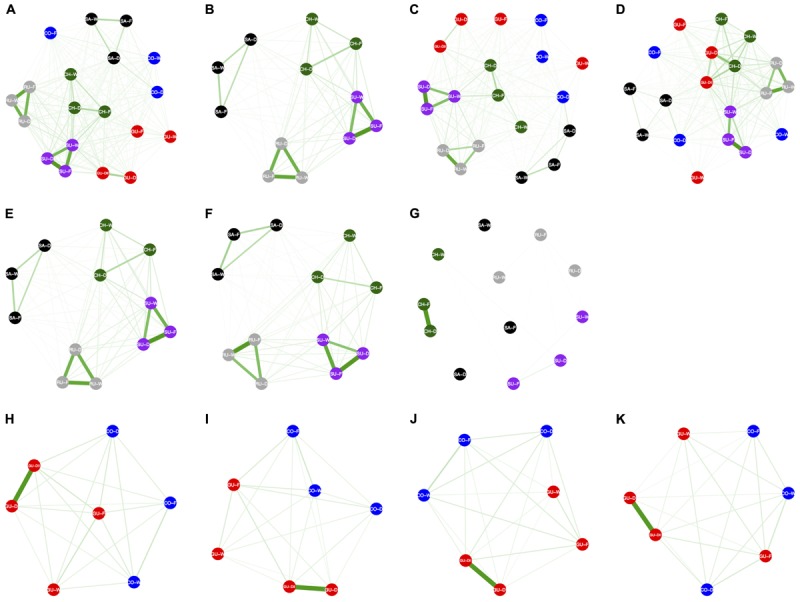
Networks depicting bidirectional gene flow patterns among populations of *Espeletia*. The width and color intensity of the green lines are proportional to the number of migrants per generation (N_e_m). The thinnest lines correspond to N_e_m values below 1. The first row of the diagrams are based on the entire SNP set including all populations **(A–D)**, while second and third rows only include the datasets for contrasting populations for localities **(E–G)** and habitats **(H–K)**, respectively. The first column of the diagrams are based on the entire SNP dataset **(A,E,H)**, while second, third, and fourth columns only include markers significantly associated with the localities **(B,F,I)**, the theoretical exposure to frost **(C,G,J)** and the theoretical soil moisture content **(D,K)**, as coded in Supplementary Table S1. The dataset that only includes contrasting populations for localities and markers significantly associated with the theoretical soil moisture content is not shown because lack of polymorphism. Allelic associations of the GBS-derived SNP markers with localities and habitats were quantified using 18 different general and mixed-effects statistical models that accounted for phylogenetic distance. Only results for the optimal models are shown (see details in Supplementary Figure S4). Localities and ecotypes are colored and coded as in Figure 1.

Estimates of migration rates (N_e_m) among populations were high and ranged from 0.3 to 7.5 for the entire dataset, and from 0.4 to 3.4 and from 0.3 to 7.5 when only contrasting populations for habitats and localities were considered, respectively. Estimated migration rates were asymmetric among these three types of datasets (Figure 5 and Table 2) with overall higher pairwise migration rates for the entire (N_e_m = 0.9 ± 0.1, CI 95%: 0.4 – 5.2, Figure 5A) and the locality-based (N_e_m = 1.2 ± 0.2, CI 95%: 0.4 – 5.8, Figure 5E) datasets than for the habitat-based dataset (N_e_m = 0.6 ± 0.1, CI 95%: 0.4 – 2.0, Figure 5H). Pairwise migration rates were higher among populations within the same locality than among populations from the same habitat from different localities for the entire (N_e_m = 3.4 ± 0.6, CI 95%: 2.9 – 4.6 vs. N_e_m = 0.7 ± 0.1, CI 95%: 0.5 – 1.5, *P*-value < 0.001, Figures 5A–D) and for the locality-based datasets (N_e_m = 3.8 ± 0.5, CI 95%: 3.3 – 4.6 vs. N_e_m = 0.6 ± 0.1, CI 95%: 0.4 – 1.0, *P*-value = 0.125, Figures 5E–G); whereas pairwise migration rates among contrasting populations for habitats did not exhibit this geographic clustering (N_e_m = 0.7 ± 0.3, CI 95%: 0.4 – 1.8 vs. N_e_m = 0.4 ± 0.1, CI 95%: 0.4 – 0.5, *P*-value = 0.002, Figures 5H–K). The datasets of alternative SNP clusters recovered the same results, except for the locality-based dataset that only included SNPs that were significantly associated with each habitat, ranked according to their theoretical exposure to frost, where the majority of pairwise migration rates were negligible except for the two Chingaza populations that were not in the cloud forest (Figure 5G). Results for theoretical soil moisture content were not possible because of a lack of polymorphism in the associated SNPs. These results were kept when considering only N_e_m estimates > 1 (Supplementary Figure S5), regarded as the minimum required for panmixia (Hartl and Clark, 2007).

**Table 2:** Average estimates of bidirectional migration rates among populations of *Espeletia*.

Population dataset	Across all populations	Among habitats within locality	Within habitats across localities
All	0.9 ± 0.1 [0.4 – 5.2]	3.4 ± 0.6 [2.9 – 4.6]	0.7 ± 0.1 [0.5 – 1.5]
Locality-based	1.2 ± 0.2 [0.4 – 5.8]	3.8 ± 0.5 [3.3 – 4.6]	0.6 ± 0.1 [0.4 – 1.0]
Habitat-based	0.6 ± 0.1 [0.4 – 2.0]	0.7 ± 0.3 [0.4 – 1.8]	0.4 ± 0.1 [0.4 – 0.5]


*Maximum likelihood estimation of the number of the number of migrants per generation (N_*e*_m) is averaged across all populations, among populations from different habitats within the same locality and among populations from the same habitat across localities. The standard error around the mean is shown after the estimate, and the 95% confidence intervals are depicted within the square brackets. Estimates are gathered from Figure 5.*

**FIGURE 6:**
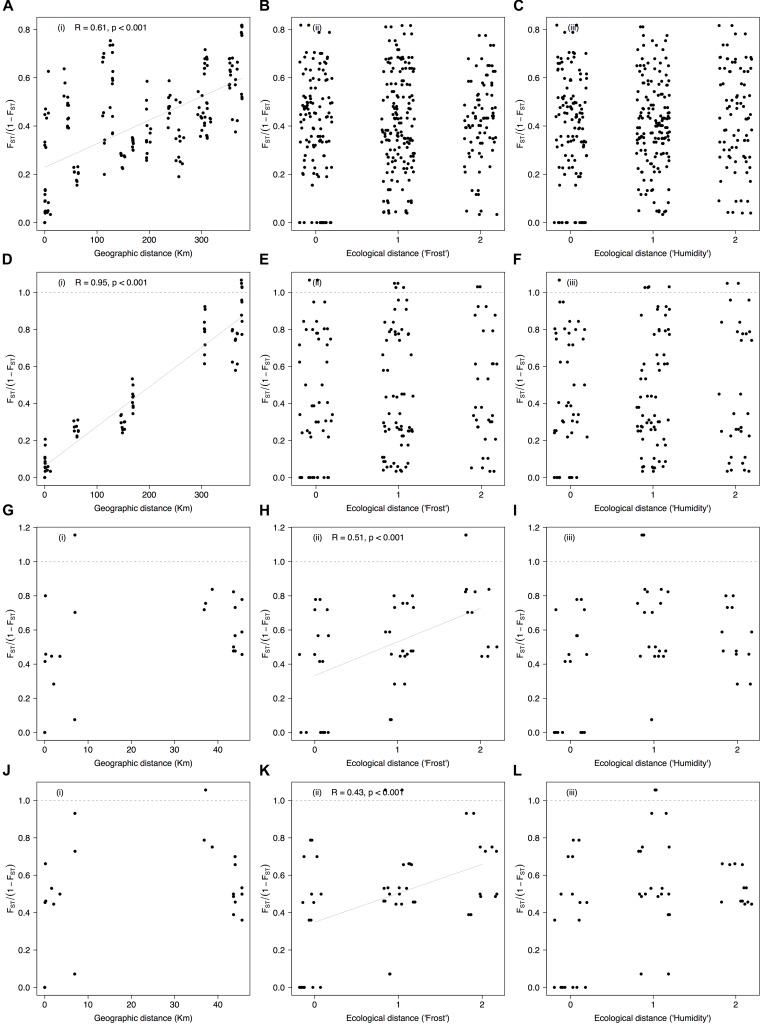
Correlations between genetic differentiation, geographic distance, and ecological distance (exposure to frost and soil moisture content) among populations of *Espeletia* growing in three different habitats and six localities. First column of diagrams show *F*_ST_/(1 – *F*_ST_) vs. geographic distance **(A,D,G,J)**, following Rousset (1997), while second and third columns show *F*_ST_/(1 – *F*_ST_) vs. ecological distances based on the theoretical (as coded in Supplementary Table S1) exposure to frost **(B,E,H,K)** and soil moisture content **(C,F,I,L)**, respectively. The first row of the diagrams are based on the entire dataset **(A–C)**, second row only include contrasting populations and markers significantly associated with the localities **(D–F)**, and third and fourth rows only include contrasting populations for habitats and markers significantly associated with the theoretical exposure to frost **(G–I)** and the theoretical soil moisture content **(J–L)**, respectively. Allelic associations of the GBS-derived SNP markers with localities and habitats were quantified using 18 different general and mixed-effects statistical models that accounted for phylogenetic distance. Only results for the optimal models are shown. Lines are displayed where Mantel tests were significant according to Table 1.

Mantel tests between genetic differentiation and geographic distance were significant when considering all populations and SNPs (average *r* = 0.59 ± 0.13, *P*-value < 0.001, Supplementary Figures S6A(i)–D(i) and Table 1). Mantel tests were also significant in the dataset enriched for contrasting localities when using the entire SNP panel (*r* = 0.95, *P*-value < 0.001, Figure 6A) and only those markers significantly associated with the localities (*r* = 0.95, *P*-value < 0.001, Figure 6D). In contrast, Mantel tests between genetic differentiation and ecological distance based on the theoretical exposure to frost were significant in the dataset with contrasting populations for habitats irrespective of the SNP set [average *r* = 0.47 ± 0.02, *P*-value < 0.001, Figures 6H,K and Supplementary Figures S6 H(ii)–K(ii)]. Mantel tests between genetic differentiation and the ecological distance based on the theoretical soil moisture content were not significant (average *r* = 0.16 ± 0.04, *P*-value > 0.05, third column in Figure 6 and Supplementary Figure S6).

## Discussion

If ecological differentiation contributed to genetic divergence in the *Espeletia* complex, then subtle and localized signals of IBE must be identifiable besides a predominant pattern of isolation-by-distance (IBD). We found strong evidence for IBD across populations despite rampant gene flow, as well as a subtle IBE trend that emerged and masked the IBD signal when we only considered contrasting populations for habitats. This finding is supported mainly by two significant results. First, migration rates were not higher among populations from the same habitat from different localities than among populations from different habitats within the same locality only for the SNP dataset enriched for habitat comparisons. Second, Mantel tests between genetic differentiation and ecological distance based on the theoretical exposure to frost, a proxy for adaptation to Páramo environments, were significant in the dataset with contrasting populations for habitats without exhibiting an, otherwise ubiquitous, relationship with the geographic distance. In other words, we have shown how IBD breaks down at the microenvironment level, allowing IBE to come into play. Mixed signatures of IBD and IBE indicate that geographical isolation and environmental heterogeneity both contribute to the spatial genetic patterns in the *Espeletia* complex. Since we were able to recover subtle and localized signals of IBE, besides a predominant pattern of IBD, there is evidence that ecological differentiation may have contributed to genetic divergence in the *Espeletia* complex. This illustrates the importance of local-scale environmental heterogeneity in keeping multiple putative adaptations and generating rapid morphological variation in a highly diverse ecosystem like the Páramo.

### *Ad hoc* Evidence of Rampant Gene Flow in the *Espeletia* Complex

The migration estimates obtained in this study indicate rampant gene flow among populations from different localities and ecotypes (caulescent populations from the cloudy forest and from wind-sheltered well-irrigated depressions, and acaulescent populations from wind-exposed drier slopes). Judging by the result that maximum nine genetic clusters spanned 19 populations (locality–habitat combinations), we can conclude that there is little evidence pointing toward an assembly of genetically well-differentiated populations fully concordant with the ecological and morphological differentiation that is observed in the *Espeletia* complex. Results from N_e_m estimates >1 are often understood as an indication of panmixia (Hartl and Clark, 2007), and here were high in the pairwise comparisons among populations from different localities and habitats. However, we conjecture that this result does not preclude finding genetic differentiation at a narrower genomic scale, for which a close reference genome would be required. For instance, particular genomic regions may exhibit signatures of local adaptation despite the fact that most of the genome freely recombines (Andrew et al., 2012; Kremer et al., 2012). Mosaics of differentiation result from contrasting signatures of divergence and gene flow that simultaneously imprint the genomic landscape (Nosil and Feder, 2011; Ellegren and Galtier, 2016). Discerning among conflicting signatures is necessary in order to depict the causes of the genome-wide patterns of geographic and ecological divergence (Cortés and Blair, 2018b; Cortés et al., 2018).

There are several reasons why gene flow could be rampant in organisms with limited seed dispersal such as *Espeletia*. First, sporadic pollen dispersal may overcome the limited seed dispersal as the mechanism for gene flow. In *Espeletia* it has been hypothesized that pollen dispersal may not be as constrained as seed dispersal (Cuatrecasas, 2013). Second, intermediate ecotypes and hybrids may act as bridges for gene flow, particularly in the *Espeletia* complex that is well known for the high incidence of natural hybridization (Rauscher, 2002; Pouchon et al., 2018). Third, episodes of geographic and habitat connectivity as part of the glacial cycling could have offered a time window for gene flow to occur across barriers, that today appear stable. Because some *Espeletia* individuals may live for several centuries (Cuatrecasas, 2013), sporadic geographic connectivity may be frequent in *Espeletia*. Finally, the absence of strong population differentiation may be due to weak reproductive barriers resulting from recent divergence. The diversification of *Espeletia* occurred in recent geological time, after the final uplift of the Northern Andes about 2.3 Myr (Pouchon et al., 2018). Gene flow within the genus clearly introduces various caveats when it comes to the reconstruction of evolutionary history. Inferring a phylogenetic tree of *Espeletia* has proven to be challenging (Rauscher, 2002; Diazgranados and Barber, 2017), despite recent progress with next-generation sequencing (NGS) technologies (Pouchon et al., 2018), likely a consequence of divergence with gene flow. The uniqueness of our study is that we coupled a coalescent reconstruction with methods that allow for admixture and reticulate evolution in order to get a better representation of the porous meta-population structure instead of limiting our sampling to a collection of single voucher specimens. In any case, the most likely explanations and consequences for the widespread gene flow in the presence of morphological and ecological differentiation remain to be studied further within the *Espeletia* complex by targeting morphologically intermediate populations as well as their putative parental lineages.

### Ecological Isolation as Driver of the Rapid Diversification in the *Espeletia* Complex

Environmental effects play a role in shaping genetic diversity and generating morphological radiations in highly heterogeneous environments (Hughes and Atchison, 2015). Radiation has traditionally been described in the light of morphological traits and ecotypes. Yet, we have framed our study in an expanded view of radiation at the genetic level (Nosil and Feder, 2011; Nosil, 2012) by exploring the drivers that shape the continuum of genomic divergence in a rapidly evolving system. This expanded view is becoming increasingly common (Gompert et al., 2014; Ravinet et al., 2017) and an explosion of literature in this topic is expected in the years to come. We have concretely contributed evidence of ecological-driven divergence in the radiation of Andean *Espeletia*. Such divergence may be a consequence of local patterns of exposure to frost, being more extreme in the wind-exposed high valley slopes than in the wind-sheltered depressions of those valleys or in the upper boundary of the cloud forest. On the other hand, soil moisture content, higher in the well-irrigated high valley depressions and in the upper boundary of the cloud forest than in the drier slopes of the valleys, does not contribute to divergence. These predictions, as any outcome of a reverse genetics approach like the GEA, must be further validated throughout experimental approaches, such as growth chamber experiments and field reciprocal transplant assays of contrasting ecotypes.

In this study we also corroborated that geographical factors are associated with divergence in *Espeletia*. Since the role of geographic isolation has already been reported and discussed *in extenso* by recent works (Diazgranados and Barber, 2017; Padilla-GonzáLez et al., 2017; Pouchon et al., 2018), here we only point out that those reports made little reference to the consequences of habitat heterogeneity (Monasterio and Sarmiento, 1991). Furthermore, we also contributed evidence that adaptive genetic divergence can be recovered despite significant IBD, as has recently been discussed by Shih et al. (2018). Despite that our sampling was enriched for contrasting ecotypes in few localities, we were still capable of recovering a predominant IBD signal, likely the result of the strength of the IBD imprint (as revealed by the observation that there were more allelic associations with the localities than with the habitats). Even though a prevalent IBD pattern seems to obscure a subtler IBE trend, both geographic and ecological isolation are likely drivers of the rapid diversification in the *Espeletia* complex, acting as the major processes diversifying the genus.

One potential caveat of our results is the disadvantage of GBS due to missing data, for example from sequence divergence (Leaché et al., 2014). Nonetheless, we were able to identify, using stringent quality filters, 1,273 GBS-derived SNP markers spread throughout the genome and with contrasting signatures of geographic- and ecological-driven divergence. It is also worth to clarify that the power to detect marker associations with localities and habitats is unlikely limited by the number of markers. The information content of a given SNP set for this type of analyses is given by the linkage disequilibrium, due to high LD in selfing plants (Carlson et al., 2004), which is higher in selfing plants (Slatkin, 2008), like *Espeletia*. SNP redundancy due to LD (Carlson et al., 2004; Slate et al., 2009; Cortés et al., 2011; Blair et al., 2012, 2013, 2018; Kelleher et al., 2012) may therefore be sufficient and adequate to perform association analyses, as done here. On the other hand, the sample size used in the association analysis may overlook the majority of associations with low effect sizes and large-effect genes that segregate at low frequency (Maher, 2008). However, genes with major effects that segregate at moderate frequencies are still recognizable, particularly given the categorical nature of the fixed factors (i.e., localities and habitats). Furthermore, since our association models accounted for phylogenetic distance, the signatures displayed on the genetic variants that are associated with localities and habitats reflect true divergence rather that confounding processes (e.g., lineage sorting), even if not all associated markers may be causal, but rather linked with causal elements, and genetic distances may be intrinsically skewed because of gene flow. Then, our result about opposing signatures of geographic and ecological isolation is robust. Our research ultimately exemplifies that regions in the world that are experiencing extremely high diversification rates, such as the alpine Netropical Páramo ecosystem (Madriñán et al., 2013), are good candidates to serve as evolutionary playgrounds for today’s scientists, just as the Galápagos Islands. By accessing the genome-wide consequences of geographic and ecological differentiation in *Espeletia*, we were able to provide evidence of ecological divergence in this genus, as has already been suggested for other highly diverse genera in the Páramo like *Loricaria* (Kolar et al., 2016) and *Lupinus* (Hughes and Eastwood, 2006; Vásquez et al., 2016). However, similar genome-wide scans should still be undertaken by using a cross-taxonomic approach (Bacon et al., 2015; Antonelli et al., 2018) in other Páramo genera that exhibit well-described high diversification rates, such as *Bartsia* (Uribe-Convers and Tank, 2015), *Diplostephium* (Vargas et al., 2017), *Hypericum* (Nürk et al., 2013), *Oreobolus* (Chacón et al., 2006; Gómez-Gutiérrez et al., 2017) and *Puya* (Jabaily and Sytsma, 2013), in order to ultimately reveal the processes that make the Páramo the fastest evolving biodiversity hotspot on earth.

## Perspectives

Besides allopatric and ecological diversification, hybridization, which was not explicitly addressed in the current sampling, is also regarded as a relevant process generating diversity in plants (Abbott et al., 2013). However, the genomic, morphological and ecological consequences of hybridization are generally not well documented across different taxa (Coyne and Orr, 2004; Payseur and Rieseberg, 2016). *Espeletia* is among the genera with a high incidence of natural hybridization and countless putative hybrids have been documented based on morphological characters (Rauscher, 2002; Cuatrecasas, 2013; Pouchon et al., 2018). Even though we have found *ad hoc* evidence of rampant gene flow, understanding the causes and consequences of hybridization in *Espeletia* and whether reticulate evolution has enhanced the diversification rate in this genus would ultimately require an in-depth ecological sampling design and an extended genotyping effort focused on hybrids and their putative parental populations.

Despite hybrids could occupy intermediate niches, persistence of populations in heterogeneous habitats that are facing changing environmental conditions is considered to be mostly mediated by phenotypic plasticity (Nicotra et al., 2010) or by adaptation from standing genetic variation by increasing the frequency of existing variants adapted to particular conditions (Bridle and Vines, 2007; Lascoux et al., 2016). Epigenetic mechanisms, those modulated by environmental factors that switch genes on and off and affect gene expression, may also alter responses to environmental variability in space and time (Bossdorf et al., 2008). Exploring the genetic architecture of ecologically relevant traits in natural surveys and field experiments, such as reciprocal transplant assays of ecotypes between habitats and space-by-time substitution trials, would help determining the relative role of plasticity and adaptation in generating morphological differentiation, as well as overall long-term adaptive potential (Barrett and Hoekstra, 2011; Valladares et al., 2014; Pacifici et al., 2017).

Some of the largest impacts of climate change are expected in highly heterogeneous alpine environments (Rumpf et al., 2018), where wind exposure and daytime vs. evening temperature fluctuations are the main drivers of vegetation composition (Körner, 2003). Temperature increases over the past decades have already led to upward migration of plants within mountains (Walther et al., 2002; Morueta-Holme et al., 2015; Steinbauer et al., 2018). However, these are mainly plant groups with short generation times (Lenoir et al., 2008) and the responses of long-living alpine plants with limited dispersal capacity are still unknown. Since *Espeletia* exhibits strong IBD and occurs in highly heterogeneous habitats, local scale variation can have important implications for their reaction to changing climatic conditions. For instance, environmental heterogeneity may provide new suitable locations for migrants within only a few meters of their current locations (Scherrer and Körner, 2011), while at the same time the populations adapted to a narrow range of conditions may respond poorly to future threats. Therefore, a key research line is to assess the evolutionary potential of *Espeletia* for coping with various types of stress that vary across habitats and that are expected to worsen at alpine ecosystems, such as frost (Wheeler et al., 2014, 2016), nutrient limitation (Sedlacek et al., 2014; Little et al., 2016), altered phenology (Cortés et al., 2014; Sedlacek et al., 2015, 2016), distorted biotic interactions (Wheeler et al., 2015), flooding and drought (well studied genetically, e.g., Cortés et al., 2012a,b, 2013; Galeano et al., 2012; Blair et al., 2016). Additionally, the evolution of the Páramo ecosystem during Andean uplift and glacial cycling presents similar conditions to current global warming and the contrasting habitats – depressions and colder and drier slopes, could be a proxy of current and future conditions. Understanding in more detail the adaptation of *Espeletia*, and other key plant groups in the Páramos, to local heterogeneous environments will thus assist with efforts to establish how future climate will impact plant populations in a highly diverse and threatened ecosystem (Vásquez et al., 2015; Pérez-Escobar et al., 2018). The contraction and expansion of populations in Páramos during past glacial fluctuations, moving up and down the mountains when it became either warmer or colder (Thuiller et al., 2008; Hazzi et al., 2018), is indicative of a general inability to adapt and a preponderant role of range shifts via migration (e.g., Bacon et al., 2018). It then remains to be seen whether the rapidly evolving and slowly dispersed *Espeletia* can keep pace with the quick rate of climate change and human expansion.

Finally, expanding our approach to other sky islands across the world’s mountains (Hoorn et al., 2018), especially in the African and Asian alpine regions, and generally to other island-like systems (Papadopoulou and Knowles, 2015; Lamichhaney et al., 2017), would help understanding how similar processes to those that that likely shaped the radiations in the neotropics have also influenced the diversity of unrelated plant groups in other regions (Condamine et al., 2018). Replicated island-like regions across the world configure an evolutionary playground to explore how radiations have taken place, as well as what are the chances for adaptation and migration to occur in response to environmental change.

## Data Accessibility

The data analysis pipeline configuration files and cleaned dataset are archived at the Dryad Digital Repository under doi: 10.5061/dryad.23bd3ds.

## Author Contributions

AC and SM designed the study. AC, LG, and SM collected the samples. AC analyzed the data with contributions from JBV and SM. AC wrote up the results with editions from the other authors.

## Conflict of Interest Statement

The authors declare that the research was conducted in the absence of any commercial or financial relationships that could be construed as a potential conflict of interest.

## Footnotes

1 http://weizhongli-lab.org/cd-hit/

2 http://bio-bwa.sourceforge.net/

3 https://github.com/ekg/freebayes#readme

## Supplementary Material

Figure S1 Inset of the population structure for the Guantiva – Chingaza – Sumapaz complex as revealed by a principal coordinates analysis (PCoA) based on 1,273 GBS-derived SNP markers. Ecotypes are labeled by different symbols as follows: caulescent populations from the cloudy forest (+), caulescent populations from wind-sheltered well-irrigated depressions (o) and acaulescent populations from wind-exposed drier slopes (), and abbreviated in the figure legend according to the habitat as forest (F), wet (W) and dry (d), respectively. Localities are identified by different colors, as in Figure 1, and abbreviated in the figure legend as follows: Chingaza (CH), Guantiva (GU), and Sumapaz (SU). The percentage of explained variation by each axis is shown within parenthesis in the label of the correspond axis.

Figure S2 Evanno’s delta *K* for the unsupervised Bayesian genetic clustering conducted in STRUCTURE with 1,273 GBS-derived SNP markers. *K* values ranged from *K* = 2 to *K* = 19. Transformed likelihoods of the graph model from Evanno et al. (2005) are shown in the vertical axis.

Figure S3 Cloudgram depicting SNAPP-based phylogenetic trees of the *Espeletia* populations analyzed in the current study using 1,273 GBS-derived SNP markers. Populations from Santurbán (SA) were used as out-groups for rooting the phylogenetic trees following Diazgranados and Barber (2017). Populations’ names are given by the combination of codes for localities and ecotypes, coded as in Figure 1.

Figure S4 Genome–environment association (GEA) analyses between the SNP markers and the localities, and between the SNP markers and the habitats. Habitats were ranked according to the theoretical exposure to frost and the theoretical soil moisture content, as coded in Supplementary Table S1. Given are eighteen QQ-plots of -log10 (*P*-value) for generalized (GLM, **A**–**C**,**G**–**I**,**M**–**O**) and mixed (MLM, **D**–**F**,**J**–**L**,**P**–**R**) linear models ran with the entire set of populations **(A–F)** or only with contrasting populations for localities **(G–L)** and for habitats **(M–R)**. Models with the locality, the theoretical exposure to frost and the theoretical soil moisture content as fixed effects are respectively shown in the first, second, and third columns. Models include as covariate the phylogenetic distance computed in SNAPP (Figure 4). All MLMs use a centered IBS kinship matrix as a random effect. The gray dashed horizontal lines mark the *P*-value thresholds after Bonferroni-correction for multiple comparisons.

Figure S5 Networks depicting bidirectional gene flow patterns leading to panmixia (N_e_m > 1) among populations of *Espeletia*. The width of the green lines is proportional to the number of migrants per generation (N_e_m) and are shown only if N_e_m > 1. First row of diagrams are based on the entire set of populations **(A–D)**, whereas second and third rows only include contrasting populations for localities **(E–G)** and for habitats **(H–K)**, respectively. First column of diagrams are based on the entire SNP dataset **(A,E,H)**, while second, third and fourth columns only include markers significantly associated with the localities **(B,F,I)**, the theoretical exposure to frost **(C,G,J)** and the theoretical soil moisture content **(D,K)**, respectively. The dataset that only includes contrasting populations for localities and markers significantly associated with the theoretical soil moisture content is not shown because lack of polymorphism (one variable site). Allelic associations were quantified as described in the legend of Figure 5. Maximum N_e_m values are shown within parenthesis in the upper left corner. Localities are identified by different colors. Populations’ names within nodes are given by the combination of codes for localities and ecotypes, colored and coded as in Figure 1.

Figure S6 Correlations between genetic differentiation, geographic distance and ecological distance (exposure to frost and soil moisture content) among populations of *Espeletia* growing in 3 different habitats and 6 localities. First column of diagrams show *F*_ST_/(1 – *F*_ST_) vs. geographic distance **(i)**, following Rousset (1997), while second and third columns show *F*_ST_/(1 – *F*_ST_) vs. ecological distances based on theoretical (as coded in Supplementary Table S1) exposure to frost **(ii)** and soil moisture content **(iii)**, respectively. First four rows of diagrams are based on the entire set of populations **(A–D)**, whereas the following three and four rows only include contrasting populations for localities **(E–G)** and for habitats **(H–K)**, respectively. First row of diagrams within these previous sets are based on the entire SNP dataset **(A,E,H)**, whereas second, third and fourth rows only include markers significantly associated (based on the optimum model among 18 different statistical models that accounted for phylogenetic distance) with the localities **(B,F,I)**, the theoretical exposure to frost **(C,G,J)** and the theoretical soil moisture content **(D,K)**, respectively. Lines are displayed where Mantel tests were significant according to Table 1.

Table S1 Identity of the 19 *Espeletia* populations used in this study and spanning 162 samples growing in 3 different habitats and 6 localities. Taxa names follow Cuatrecasas (2013). Populations’ abbreviations are given by the combination of codes for localities and ecotypes. Localities are coded as follows: Chingaza (CH), Cocuy (CO), Guantiva (GU), Santurbán (SA), Sumapaz (SU) and Ruíz (RU). Ecotypes are coded as follows: caulescent populations from the cloudy forest (F), caulescent populations from wind-sheltered well-irrigated depressions (W) and acaulescent populations from wind-exposed drier slopes (D). Under “Ecology” habitats are coded using an ordinal scale based on the theoretical exposure to frost and the theoretical soil moisture content.

Table S2 Population structure from assignment tests in STRUCTURE based on 1,273 variable SNP markers. Probabilities are shown for the optimum *K*-value (*K* = 9, Supplementary Figure S2) according to Evanno et al. (2005), the PCoA (Figure 2) and visual inspection. Populations are abbreviated as in Supplementary Table S1.
