# Supplementary figures and images for: On the Causes of Rapid Diversification in the Páramos: Isolation by Ecology and Genomic Divergence in Espeletia
Source: Front Plant Sci. 2018 Dec 3;9:1700. doi: 10.3389/fpls.2018.01700 (PMC6294130; doi:10.3389/fpls.2018.01700)

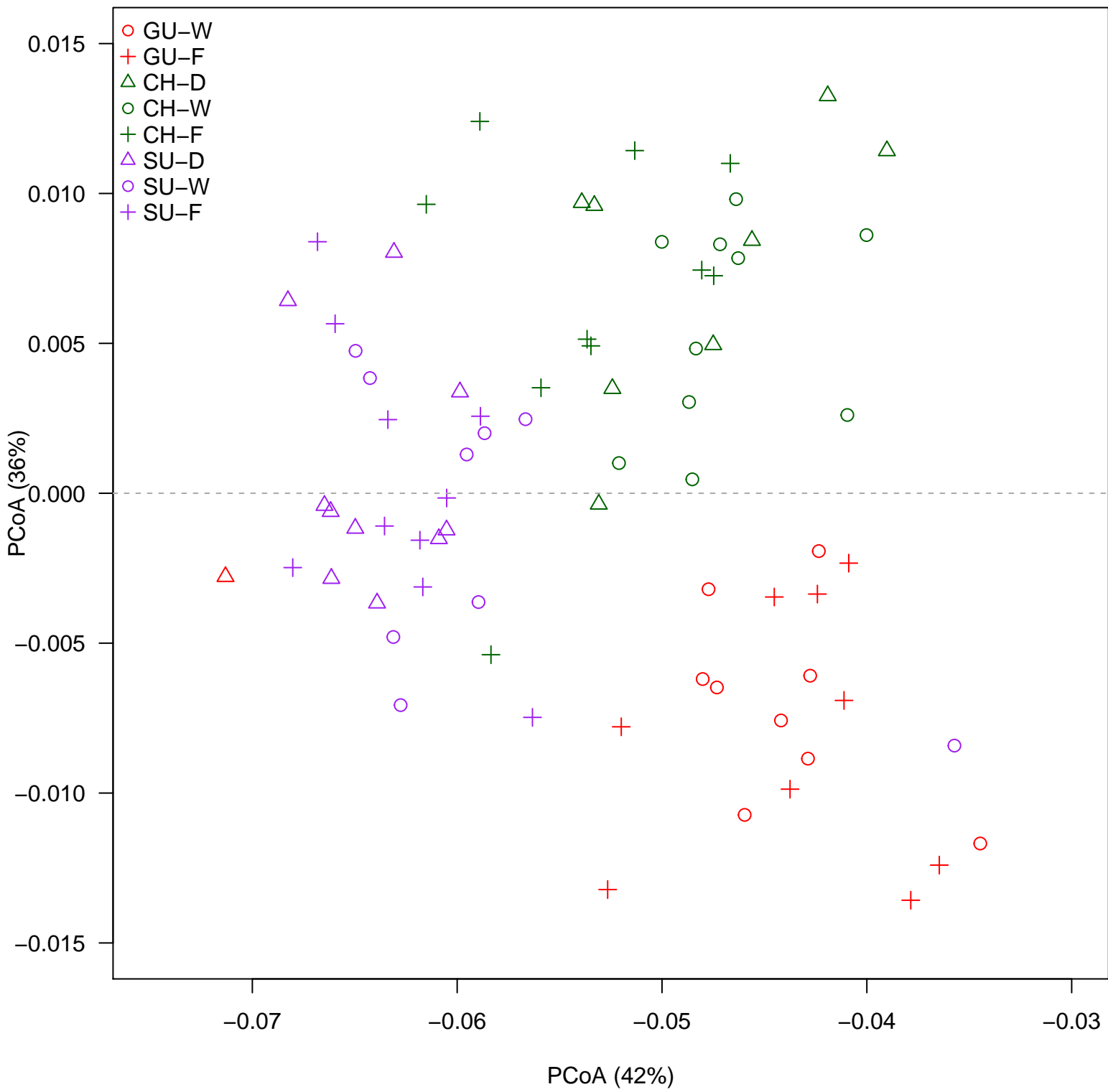

Supplement: Figure S1 — Inset of the population structure for the Guantiva – Chingaza – Sumapaz complex as revealed by a principal coordinates analysis (PCoA) based on 1,273 GBS-derived SNP markers. Ecotypes are labeled by different symbols as follows: caulescent populations from the cloudy forest (+), caulescent populations from wind-sheltered well-irrigated depressions (o) and acaulescent populations from wind-exposed drier slopes (), and abbreviated in the figure legend according to the habitat as forest (F), wet (W) and dry (d), respectively. Localities are identified by different colors, as in Figure 1, and abbreviated in the figure legend as follows: Chingaza (CH), Guantiva (GU), and Sumapaz (SU). The percentage of explained variation by each axis is shown within parenthesis in the label of the correspond axis. [file Data_Sheet_1.PDF]

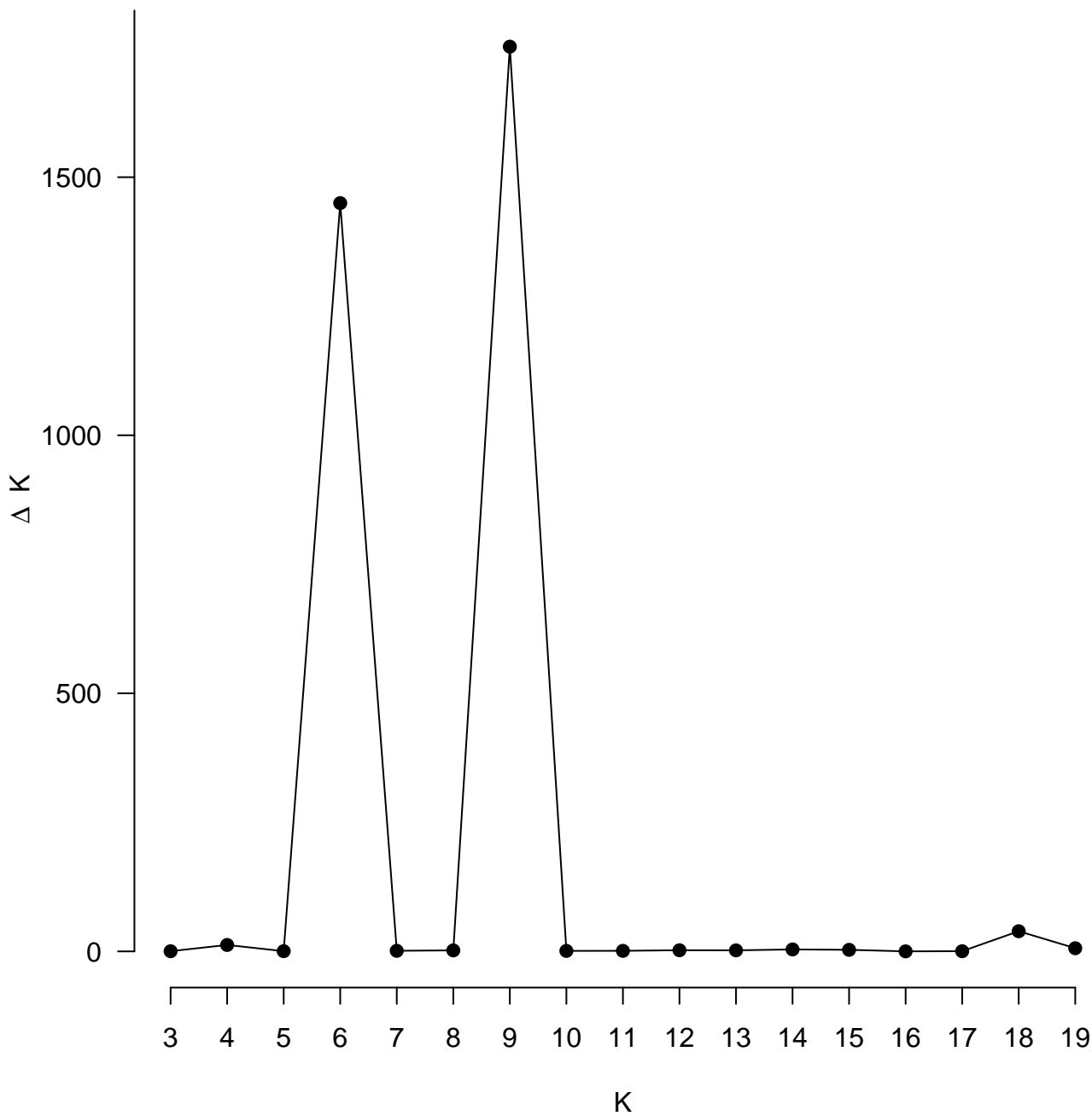

Supplement: Figure S2 — Evanno’s delta K for the unsupervised Bayesian genetic clustering conducted in STRUCTURE with 1,273 GBS-derived SNP markers. K values ranged from K = 2 to K = 19. Transformed likelihoods of the graph model from Evanno et al. (2005) are shown in the vertical axis. [file Data_Sheet_2.PDF]

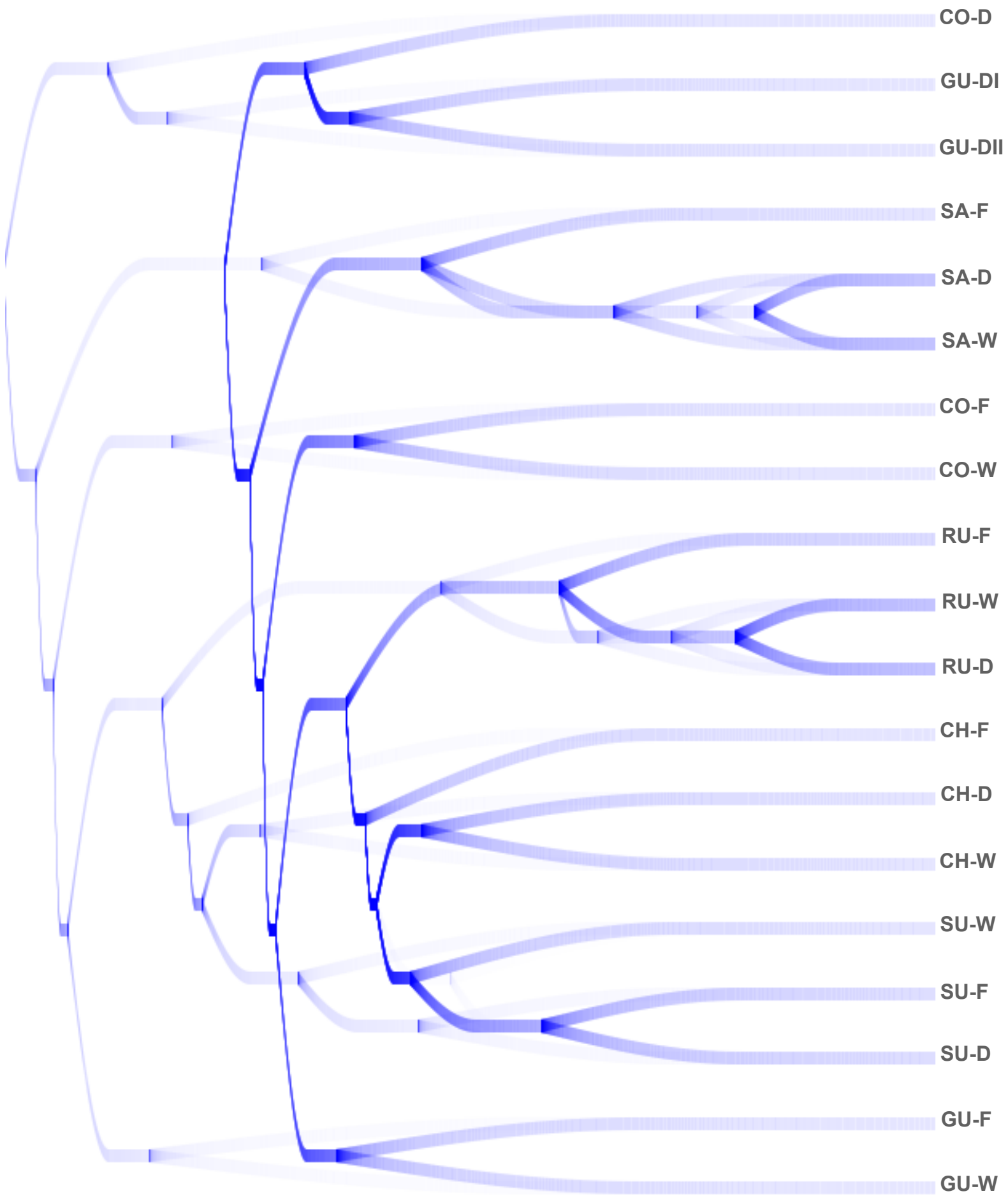

Supplement: Figure S3 — Cloudgram depicting SNAPP-based phylogenetic trees of the Espeletia populations analyzed in the current study using 1,273 GBS-derived SNP markers. Populations from Santurbán (SA) were used as out-groups for rooting the phylogenetic trees following Diazgranados and Barber (2017). Populations’ names are given by the combination of codes for localities and ecotypes, coded as in Figure 1. [file Data_Sheet_3.PDF]

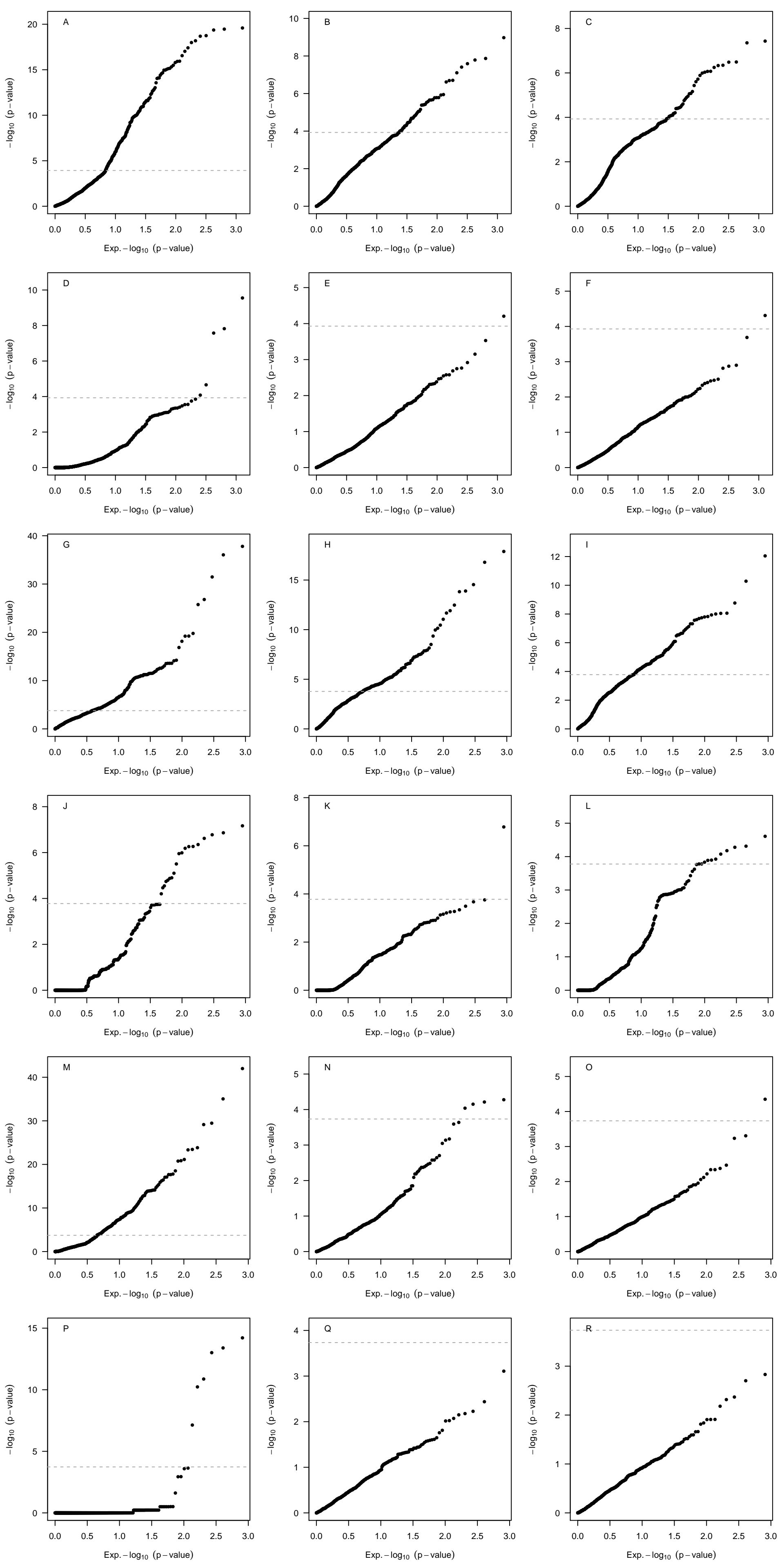

Supplement: Figure S4 — Genome–environment association (GEA) analyses between the SNP markers and the localities, and between the SNP markers and the habitats. Habitats were ranked according to the theoretical exposure to frost and the theoretical soil moisture content, as coded in Supplementary Table S1. Given are eighteen QQ-plots of -log10 (P-value) for generalized (GLM, A–C,G–I,M–O) and mixed (MLM, D–F,J–L,P–R) linear models ran with the entire set of populations (A–F) or only with contrasting populations for localities (G–L) and for habitats (M–R). Models with the locality, the theoretical exposure to frost and the theoretical soil moisture content as fixed effects are respectively shown in the first, second, and third columns. Models include as covariate the phylogenetic distance computed in SNAPP (Figure 4). All MLMs use a centered IBS kinship matrix as a random effect. The gray dashed horizontal lines mark the P-value thresholds after Bonferroni-correction for multiple comparisons. [file Data_Sheet_4.PDF]

A (7.5)

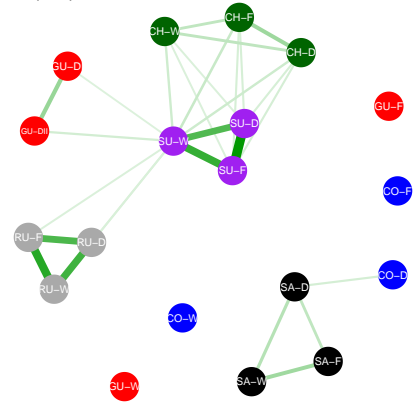

B (7.3)

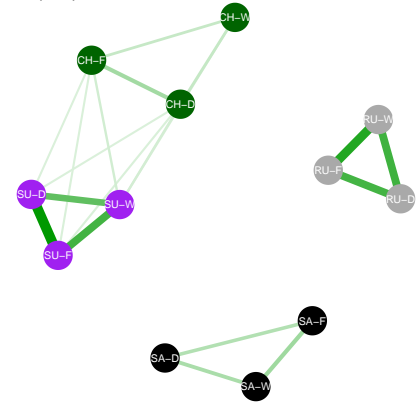

C (11)

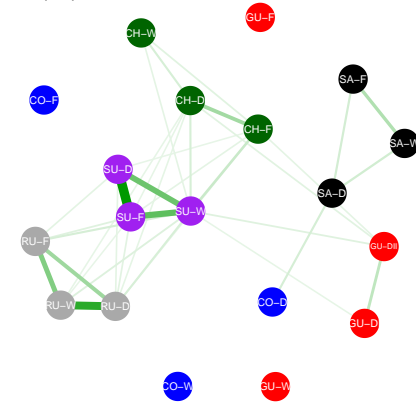

D (11.9)

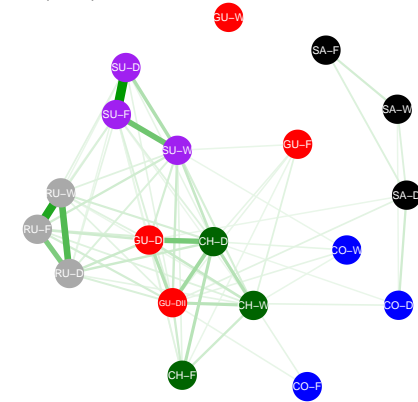

E (7.3)

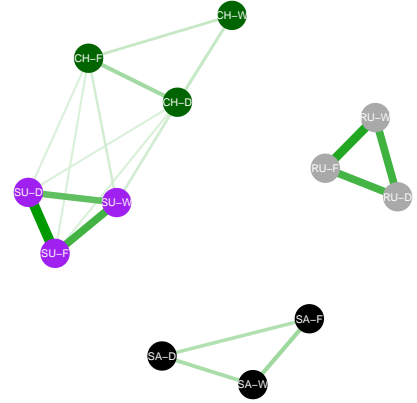

F (7.5)

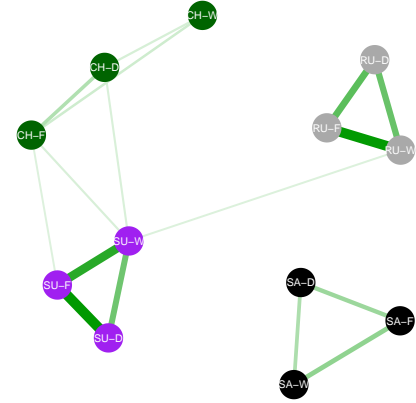

G (240.6)

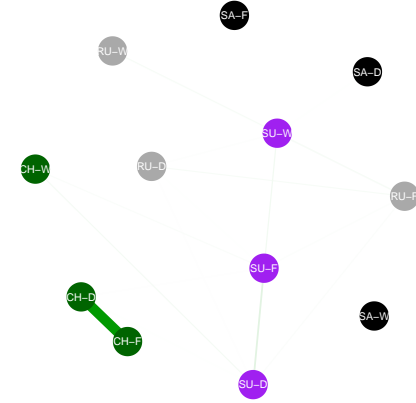

H (3.4)

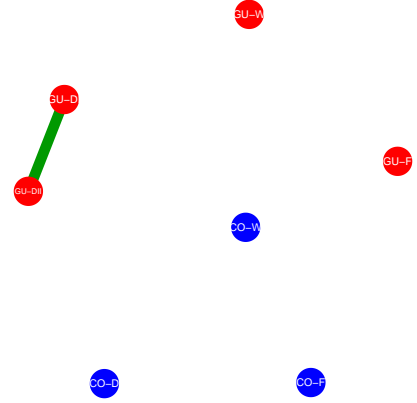

I (3.5)

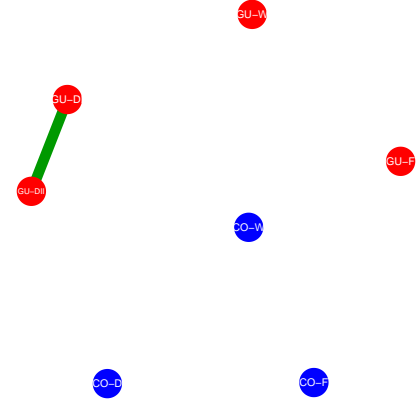

J (3.3)

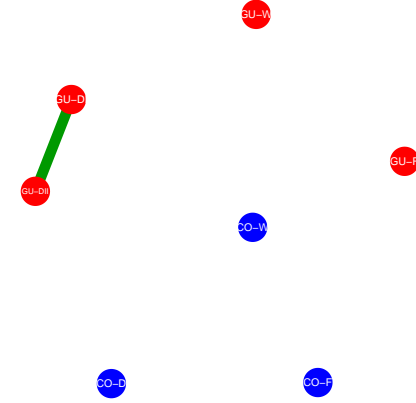

K (3.5)

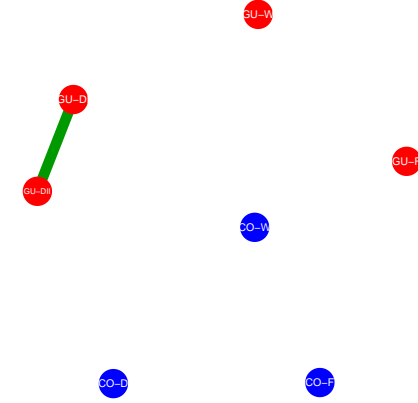

Supplement: Figure S5 — Networks depicting bidirectional gene flow patterns leading to panmixia (Nem > 1) among populations of Espeletia. The width of the green lines is proportional to the number of migrants per generation (Nem) and are shown only if Nem > 1. First row of diagrams are based on the entire set of populations (A–D), whereas second and third rows only include contrasting populations for localities (E–G) and for habitats (H–K), respectively. First column of diagrams are based on the entire SNP dataset (A,E,H), while second, third and fourth columns only include markers significantly associated with the localities (B,F,I), the theoretical exposure to frost (C,G,J) and the theoretical soil moisture content (D,K), respectively. The dataset that only includes contrasting populations for localities and markers significantly associated with the theoretical soil moisture content is not shown because lack of polymorphism (one variable site). Allelic associations were quantified as described in the legend of Figure 5. Maximum Nem values are shown within parenthesis in the upper left corner. Localities are identified by different colors. Populations’ names within nodes are given by the combination of codes for localities and ecotypes, colored and coded as in Figure 1. [file Data_Sheet_5.PDF]

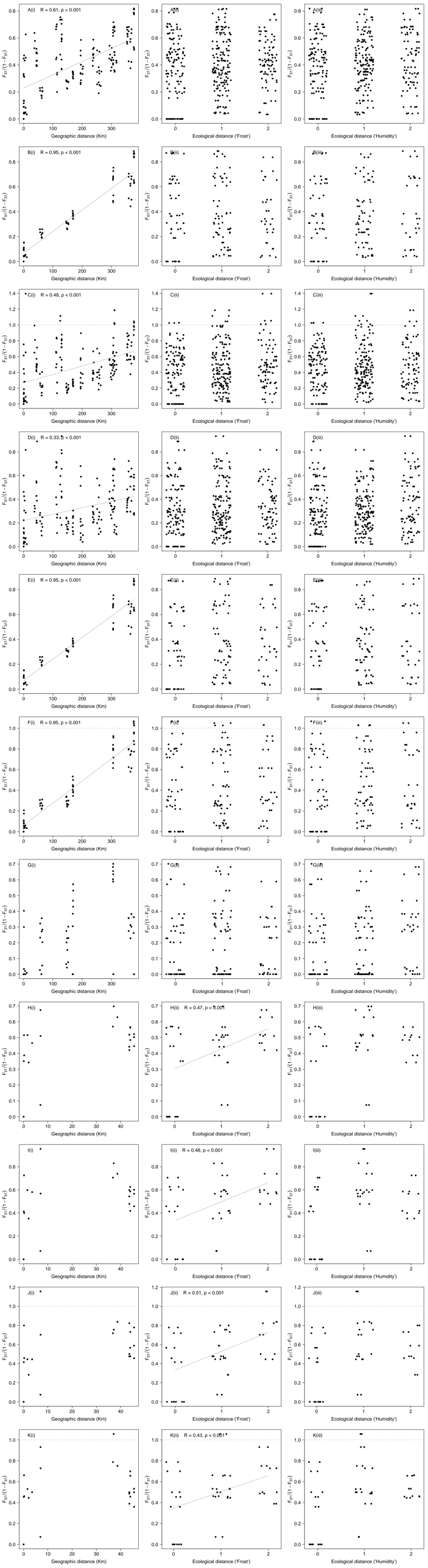

Supplement: Figure S6 — Correlations between genetic differentiation, geographic distance and ecological distance (exposure to frost and soil moisture content) among populations of Espeletia growing in 3 different habitats and 6 localities. First column of diagrams show FST/(1 – FST) vs. geographic distance (i), following Rousset (1997), while second and third columns show FST/(1 – FST) vs. ecological distances based on theoretical (as coded in Supplementary Table S1) exposure to frost (ii) and soil moisture content (iii), respectively. First four rows of diagrams are based on the entire set of populations (A–D), whereas the following three and four rows only include contrasting populations for localities (E–G) and for habitats (H–K), respectively. First row of diagrams within these previous sets are based on the entire SNP dataset (A,E,H), whereas second, third and fourth rows only include markers significantly associated (based on the optimum model among 18 different statistical models that accounted for phylogenetic distance) with the localities (B,F,I), the theoretical exposure to frost (C,G,J) and the theoretical soil moisture content (D,K), respectively. Lines are displayed where Mantel tests were significant according to Table 1. [file Data_Sheet_6.PDF]
